# Editorial: The role of one-carbon metabolism in cancer progression, therapy, and resistance

**DOI:** 10.3389/fonc.2023.1286790

**Published:** 2023-09-21

**Authors:** Abhimanyu Thakur, Xin Hu, Erhu Zhao, Chunwan Lu, Yanqing Liu, Yashika Rustagi, Kui Zhang

**Affiliations:** ^1^ Pritzker School of Molecular Engineering, Ben May Department for Cancer Research, University of Chicago, Chicago, IL, United States; ^2^ State Key Laboratory of Resource Insects, Southwest University, Chongqing, China; ^3^ Cancer Center, Medical Research Institute, Southwest University, Chongqing, China; ^4^ School of Life Sciences, Tianjin University, Tianjin, China; ^5^ Herbert Irving Comprehensive Cancer Center, Columbia University, New York, NY, United States; ^6^ Department of Medical Oncology, Dana Farber Cancer Institute, Boston, MA, United States

**Keywords:** folic acid, cancer diagnosis and treatment, nucleic acid synthesis, chemotherapy, metabolic pathways

Folate metabolism is an essential metabolic process utilized by bacteria, yeast, plants, and animals, and is responsible for activating and transferring one-carbon (1C) units for biosynthetic processes, such as the synthesis of purines and thymidine, as well as remethylating of homocysteine. Animals require dietary folate, without it, adults can develop anemia, and fetuses can suffer from neural tube defects. Neural tube defects can be mild to severe and, in the most serious cases, can lead to fetal loss or partial paralysis of the legs. The inhibition of folate metabolism then, due to its role in nucleic acid synthesis, can impede cellular proliferation. Consequently, antibiotics and chemotherapeutic agents have been developed to specifically target folate metabolism, such as the combination of sulfamethoxazole and trimethoprim or methotrexate and pemetrexed (1–4).

In the context of folate chemistry, folate molecules are small molecules composed of 3 distinct chemical moieties; a pteridine ring (which may be reduced or oxidized), a para-aminobenzoic acid (PABA) linker, and polyglutamate tail that anchors the molecule inside a cell. The form with the lowest energy is known as tetrahydrofolate (THF), which is the biologically active form of folate and is present as 5-methyl-THF in humans. To be used, folic acid (vitamin B9) must be reduced to dihydrofolate and then THF to enter the folate cycle. THF acts as a carrier for 1 C units, which are covalently bound to the 5-position nitrogen atom from the pteridine ring and the 10-position nitrogen atom from the PABA moiety. These 1C units can then be manipulated and exchanged between different oxidation states, namely 5,10-methylene-THF, 5-methyl-THF, 10-formyl-THF, and 5-formyl-THF, where the last does not serve any biosynthetic purpose but acts as a storehouse instead. The primary source of 1C units is 5, 10-methylene-THF, which can be formed from amino acids, namely serine, glycine, dimethyl glycine, and methyl glycine. In conclusion, folates and 1C units play an essential role in metabolic processes, both in the mitochondrial matrix and the cytosol (5, 6). **Figure 1** shows the schematic representation of the metabolic cycle of THF and the C1 metabolic pathway.

**Figure 1:**
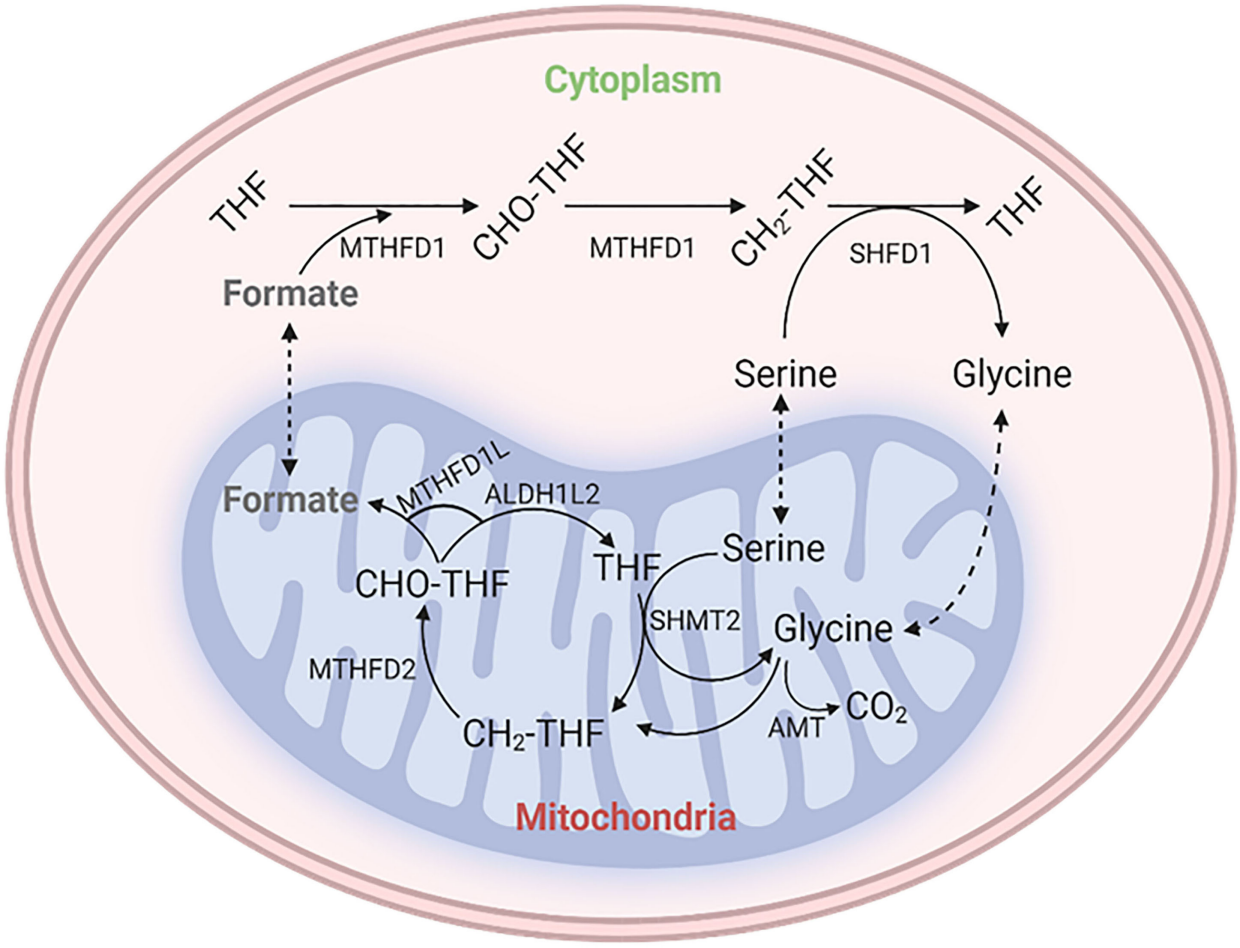
Schematic diagram of the metabolic cycle of tetrahydrofolate (THF) and the one-carbon (C1) metabolic pathway. The representation depicts the different reactions involved in the metabolic cycle of THF and the different sources and sinks of one-carbon units. THF is a coenzyme that is involved in the transfer of one-carbon units in many metabolic reactions. The C1 metabolic pathway is responsible for the generation and utilization of one-carbon units.

Researchers have studied the dietary requirement of folate in both development and adulthood, primarily because of its major role in nucleic acid synthesis for cellular proliferation. Recently, the roles of specific folate-utilizing enzymes in growth, development, fetal development, immune proliferation, and tissue homeostasis have been elucidated. Mouse embryonic stem cells (ESCs) have a rapid doubling period of just 4-5 hours, which can introduce unique metabolic stressors. Through investigations of threonine-deprived ESCs, it was discovered that the glycine route from Threonine Dehydrogenase (TDH) was paramount to both DNA and methionine synthesis. Without threonine, the cells cannot be viable, demonstrating a crucial dependence on glycine-sourced cytosolic 1C units. Astonishingly, the TDH reaction additionally creates the power meter, Acetyl-CoA, in a delicate balance, meaning threonine withdrawal can drastically affect histone modification due to its control of SAM and Acetyl-CoA levels. Restoration of the effects of a threonine-deprived state is achievable through pyruvate as an Acetyl-CoA source combined with external 1C donors such as glycine, dimethylglycine, and betaine (3).

Various studies have demonstrated the role of 1C metabolism in cancer progression. For example, the study by Montal et al. revealed that phosphoenol pyruvate carboxykinase (PEPCK) plays a pivotal role in regulating central carbon metabolism and promoting cancer cell growth (7). A recent study conducted by Sayin et al. examined the effect of the activation of the NRF2 transcription factor and its role in skewing central carbon metabolism in cancer. Their findings indicated that NRF2 activation caused an imbalance in the essential metabolism pathways that regulate the synthesis of macromolecules from small molecules such as glucose. This imbalance is thought to be a key factor in the pathogenesis of cancer (8). The findings of the Suzuki et al. study suggest that 1C metabolism-related gene polymorphisms may interact with folate intake to affect the risk of breast cancer (9). Labuschagne et al. investigated the role of serine and glycine in nucleotide synthesis and cancer cell proliferation. They found that cancer cells preferentially consumed serine over glycine, and that this was essential for nucleotide synthesis and cell proliferation (10). Apparently, targeting 1C metabolism is crucial in chemotherapy (3).

Liu et al. recently conducted a systematic review aimed at examining the advances in utilizing highly active 1C metabolism in cancer diagnosis, treatment, and drug resistance. The initial literature search identified eight randomized controlled trials (RCTs) to be further evaluated. Based on the Preferred Reporting Items for Systematic Reviews and Meta-Analyses (PRISMA) protocol, these selected studies were then assessed for their quality by The Grading of Recommendations Assessment, Development, and Evaluation (GRADE) approach, which yielded interesting findings. The review concluded that metabolites such as folic acid could potentially detect different types of cancer. Additionally, they found that the metabolic pathways that control 1C metabolism can also induce tumorigenesis and DNA methylation, which can drastically affect the effectiveness of drug treatments.

Aoki et al. explored the relationship between methionine dependence and malignancy in cancer cells, known as the Hoffman Effect. They used methionine-addicted osteosarcoma cells and their methionine-independent revertant cells to investigate the effects. Through various assays, they found that the methionine-addicted cells had higher migration and invasion capabilities, larger tumors, and increased metastatic potential when injected into mice. These cells also showed an epithelial-mesenchymal phenotype and specific changes in histone methylation patterns. These findings suggest that targeting methionine addiction could be a potential strategy to improve sarcoma therapy by reducing oncogenic phenotypes. This research provides valuable insights into the role of methionine in cancer cell behavior and highlights its potential as a therapeutic target. In this Research Topic, a review article by Zhang et al. explained the potential of serine-associated 1C metabolic reprogramming as a novel anti-cancer approach. Gao et al. have discussed the future prospective therapy for anaplastic thyroid carcinoma in the context of immunotherapy or targeted therapy.

Overall, this Research Topic of articles will pave the way for future research pertaining to delineating the role of 1C metabolism in cancer.

## Author contributions

AT: Conceptualization, Writing – original draft. XH: Writing – review & editing. EZ: Writing – review & editing. CL: Writing – review & editing. YL: Writing – review & editing. YR: Writing – review & editing. KZ: Writing – review & editing.
